# P-1814. Say it Ain't Steno - Impact of a Microbiology Nudge Comment on Treatment of Respiratory Colonization

**DOI:** 10.1093/ofid/ofae631.1977

**Published:** 2025-01-29

**Authors:** Stormmy Boettcher, Rachel M Kenney, Christen J Arena, Amy Beaulac-Harris, Robert Tibbetts, Anita Shallal, Geehan Suleyman, Michael Veve

**Affiliations:** Henry Ford Hospital, Royal Oak, Michigan; Henry Ford Hospital, Royal Oak, Michigan; Eugene Applebaum College of Pharmacy and Health Sciences, Wayne State University and Henry Ford Health, Royal Oak, Michigan; Henry Ford West Bloomfield Hospital, Henry Ford Health, West Bloomfield, Michigan; Henry Ford Health, Detroit, Michigan; Henry Ford Health, Detroit, Michigan; Henry Ford Health, Detroit, Michigan; Henry Ford Health, Detroit, Michigan

## Abstract

**Background:**

*Stenotrophomonas maltophilia* (SM) is a known colonizer of the respiratory tract, in which treatment is not required. Microbiological comment “nudges” have been successful as passive stewardship interventions. The study objective was to describe the effect of a targeted SM respiratory culture nudge on antibiotic use in patients with colonization.

**Figure:**
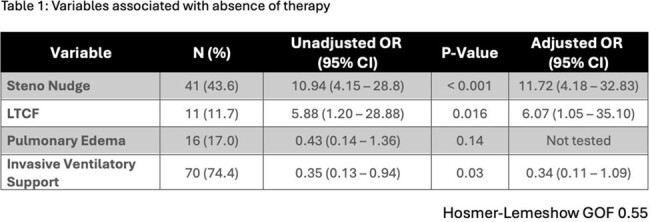

**Methods:**

IRB approved quasi-experiment of adult patients with a SM respiratory culture between 01/01/2022-01/27/2023 (pre-nudge) and 03/27/2023-12/31/2023 (post-nudge). Patients with criteria for active community/hospital/ventilator-acquired pneumonia or on targeted antibiotics prior to culture were excluded. Nudge comment implemented 2/2023: “*S. maltophilia* is a frequent colonizer of the respiratory tract. Clinical correlation for infection is required. Colonizers do not require antibiotic treatment.” The primary outcome was absence of SM therapy; secondary outcomes were SM therapy > 72 hours, hospital and ICU length of stay (LOS), and in-hospital all-cause mortality. Safety outcomes included antibiotic-related adverse events.

**Results:**

94 patients were included: 53 (56.4%) pre- and 41 (43.6%) post-nudge. Most patients were men (53, 56.4%), had underlying lung disease (61, 64.8%), and required invasive ventilatory support (70, 74.5%). 11 (11.7%) patients were admitted from a long-term care facility. The absence of SM therapy was observed in 13 (23.1%) pre- vs 32 (78.0%) post-nudge patients (P< 0.001). There were no differences in SM therapy > 72 hours (36/40 [90%] vs. 8/9 [88.9%], P=1.0), mortality (11 [20.8%] vs. 7 [17.5%], P=0.69), median (IQR) hospital LOS (24 [10-49] vs. 16 [8-29]), P=0.37), and median (IQR) ICU LOS [15 [2-35] vs. 11 [3-25], P=0.40) between pre- and post-nudge groups, respectively. Safety outcomes of patients treated > 72 hours (n=41); elevated SCr 12 (29.3%), fluid overload 18 (43.9%), hyponatremia 17 (41.5%), and hyperkalemia 5 (12.2%). After adjustment for confounders, post-nudge was associated with 11-fold increased odds of the absence of SM therapy (Table 1).

**Conclusion:**

A targeted SM nudge was associated with a reduction in treatment of SM colonization. Patient outcomes, including length of stay and all-cause mortality, were comparable between the two groups.

**Disclosures:**

**Rachel M. Kenney, PharmD, BCIDP**, Medtronic Inc: Spouse is an employee, stockholder

